# Structural insights into the nanomolar affinity of RING E3 ligase ZNRF1 for Ube2N and its functional implications

**DOI:** 10.1042/BCJ20170909

**Published:** 2018-05-09

**Authors:** Adaitya Prasad Behera, Pritam Naskar, Shubhangi Agarwal, Prerana Agarwal Banka, Asim Poddar, Ajit B. Datta

**Affiliations:** Department of Biochemistry, Bose Institute, P-1/12 CIT Scheme VIIM, Kolkata 700054, India

**Keywords:** E3 ligase activity, isothermal titration calorimetry, Ube2N, ubiquitin E3 ligase ZNRF1, ubiquitins

## Abstract

RING (Really Interesting New Gene) domains in ubiquitin RING E3 ligases exclusively engage ubiquitin (Ub)-loaded E2s to facilitate ubiquitination of their substrates. Despite such specificity, all RINGs characterized till date bind unloaded E2s with dissociation constants (*K*_d_s) in the micromolar to the sub-millimolar range. Here, we show that the RING domain of E3 ligase ZNRF1, an essential E3 ligase implicated in diverse cellular pathways, binds Ube2N with a *K*_d_ of ∼50 nM. This high-affinity interaction is exclusive for Ube2N as ZNRF1 interacts with Ube2D2 with a *K*_d_ of ∼1 µM, alike few other E3s. The crystal structure of ZNRF1 C-terminal domain in complex with Ube2N coupled with mutational analyses reveals the molecular basis of this unusual affinity. We further demonstrate that the ubiquitination efficiency of ZNRF1 : E2 pairs correlates with their affinity. Intriguingly, as a consequence of its high E2 affinity, an excess of ZNRF1 inhibits Ube2N-mediated ubiquitination at concentrations ≥500 nM instead of showing enhanced ubiquitination. This suggests a novel mode of activity regulation of E3 ligases and emphasizes the importance of E3-E2 balance for the optimum activity. Based on our results, we propose that overexpression-based functional analyses on E3 ligases such as ZNRF1 must be approached with caution as enhanced cellular levels might result in aberrant modification activity.

## Introduction

Post-translational modification of proteins by conjugation of ubiquitin (Ub) plays crucial roles in almost all subcellular processes in eukaryotes including intracellular signaling, cell cycle progression, transcription activation, and proteostasis [1,2]. In spite of having such diverse functional consequences, all ubiquitination reactions proceed via a cascade of enzymatic steps requiring sequential actions of activating E1s, conjugating E2s, and E3s. E3 ligases catalyze the final step in this cascade by promoting the transfer of activated Ubs from E2∼Ub thioester intermediates to specific substrates or to the growing ends of substrate-linked Ub chains [3]. HECT/RBR E3s achieve this via formation of E3∼Ub intermediates, whereas RING/U-box E3s facilitate a direct delivery of activated Ubs from E2∼Ub conjugates to acceptor amine groups. Most of the E3s belong to the RING (Really Interesting New Gene) class and it is estimated that human proteome contains more than 600 such active E3s, making them one of the largest group of enzymes apart from the kinases.

RING E3s are characterized by the presence of a 30–100 residue long RING domain that chelates two Zn^2+^ ions in a cross-braced arrangement, whereas the U-box variants contain a structurally similar RING-like fold but lack the Zn atoms [4]. Structural, biochemical, and genetic studies have equivocally established the role of the conserved RING domain in E2 binding and catalysis [5–7]. Biochemical analyses on multiple E3s further revealed that RING E3s bind E2s considerably weakly with dissociation constants in the 1–150 µM range [6,8]. Such poor affinities often hinder detailed characterization of E3 : E2 interaction using available experimental techniques. Weak binding also makes it difficult to determine E2 partner(s) of a novel E3 ligase by commonly employed methods such as co-immunoprecipitation. Nonetheless, consistent with their weak affinities, crystal structures of all E3 : E2 or E3 : E2∼Ub complexes failed to reveal significant structural alteration in any of the proteins upon binding. The ∼600 Å^2^ interaction interface is also conserved across most E3 : E2 pairs. In the E3s, the binding surface is constituted by the ‘spacer’ regions between the Zn^2+^-chelating residues, while three distinct sequentially noncontiguous E2 regions, namely helix1 (E2-α1), loop 4 connecting β-sheets 3 and 4 (E2-L4), and loop 7 connecting the short 3_10_ helix with the helix2 (E2-L7), contribute towards the RING interaction. RING domains, however, do not bind all the ∼38 E2s indiscriminately; rather, they confer E2 selectivity by specifically interacting with only a subset of E2s in cells. RBR class of E3s also utilizes one of their RING domains to bind E2∼Ub similar to other RING-containing E3s. But, in contrast with the classical RING E3 ligases, they utilize their catalytic Cys to receive the activated Ub from the E2∼Ub before delivering it to the final destination [9].

Not all E3s interact with their cognate E2s with weak affinities. E3 ligases such as Rad18, gp78, and cue1p have been reported to bind their respective E2 partners, namely Rad6, Ube2G2, and Ubc7p with dissociation constants in the nanomolar range [10–12]. Interestingly, all of these E3s contain additional non-RING elements to engage the E2s apart from the canonical RING domains. These non-RING elements not only enhance the overall affinity between such E3:E2 pairs but also modulate their ubiquitination activity and processivity. For example, R6BD in Rad18 restricts the E2 to mono-ubiquitination despite being capable of chain synthesis [11]. High-affinity E2 : E3 interactions may alternatively involve extended termini found in certain E2s as demonstrated by the cdc34-CRL pair, where the acidic C-terminus of cdc34 interacts with a basic cleft in the E3 thereby increasing the processivity of the E2–E3 complex [13].

Besides their functional importance, high-affinity interactions between E3s and E2s allow identification of such pairs without any ambiguity. One such study carried out by Hoxhaj et al. [14] found that RING E3 ligase ZNRF1 co-purifies with Ube2N from transiently transfected HEK293 cells in stoichiometric amounts. This E3 ligase was first identified as one of the gene products up-regulated in Schwann cells upon peripheral nerve injury and contains a Zinc-finger (ZnF) domain juxtaposed to its C-terminal RING domain [15]. ZNRF1 is found to be an essential E3 ligase for development/survival and its deletion leads to embryonic lethality in mice [14]. Though the precise cellular roles of ZNRF1 remain to be deciphered, it has been found to co-localize with tubulin in cells and ubiquitinates protein kinase B (Akt1), glutamine synthase, and the α subunit of Na^+^/K^+^ pump [16–18]. Recent studies further implicate ZNRF1 in modulating inflammation via ubiquitination of caveolin-1 establishing the importance of this protein in immune response [19]. Besides Ube2N, ZNRF1 also co-elutes with minor amounts of Ube2Ds and Ube2W suggesting high E2 affinity of this E3 ligase [14]. We set forth to understand the molecular details behind the unusual E2 affinity of ZNRF1 using binding experiments and structural studies. Our results reveal that despite having a canonical E3 : E2 interaction, mediated exclusively by the RING domain, ZNRF1/Ube2N interface is ‘tuned’ at the molecular level to achieve unprecedented nanomolar affinity. We further observe that though ZNRF1 at moderate concentrations tends to interact with its preferred partner Ube2N, an increase in its concentration inhibits Ube2N-mediated ubiquitination and engages E2s such as Ube2D2. Our results, therefore, provide with the first example of such high-affinity interaction between a RING domain and an E2 enzyme and indicate that perturbation of the concentration of an E3 ligase can potentially alter the downstream processes in an undesirable manner leading to spurious outcomes.

## Materials and methods

### Cloning, mutagenesis, and expression of proteins

PCR-amplified ORFs corresponding to ZNRF1 full-length, ZNRF1^CTD^ (residues 139–227), and ZNRF1^RING^ (residues 166–227), were cloned into pETSUMO2 vector [20] using the Infusion HD Cloning Kit (Clontech, Inc., U.S.A.) and expressed as fusion proteins containing N-terminal 6xHis–SUMO2 tag. In addition to the N-terminal tag, full-length ZNRF1 also contained a non-cleavable C-terminal strep-tag II for the ease of purification. All other constructs of the E2s, E3s, Ub, and 6xHis-Uba1 (E1) were already available in the laboratory and have already been described [21]. All the E2 mutants were generated by the PCR-based site-directed mutagenesis protocol using Kod DNA polymerase (Toyobo Life Sciences, Japan) and DpnI (New England Biolabs, U.S.A.). All clones were confirmed by DNA sequencing using Applied Biosystems sequencers (3130XL and 3500XL).

Proteins were expressed in *Escherichia coli* Rossetta2 (DE3) cells (Novagen, Inc., U.S.A.) in M9ZB media with 0.5% glycerol supplemented with antibiotics. Cells cultured at 37°C to OD_590 nm_ ≈ 1.0–2.0 were induced with 0.5 mM of isopropyl-1-thio-β-d-galactopyranoside (IPTG) and incubated at 16°C at 240 rpm for 16–18 h. For all ZNRF1 variants, the cultures were grown at 37°C to OD_590 nm_ ≈ 2.0–2.5, followed by a ‘cold shock’ in an ice-water bath for 60 min. Expression of soluble recombinant E3 samples was then achieved at 16°C at 240 rpm for ∼16 h with the addition of 0.5 mM of IPTG along with 40 µM of ZnSO_4_. Cells were harvested by centrifugation and pellets were stored at −80°C until purifications were attempted.

### Purification of proteins

Purification of Ub, E1, and all the E2s was carried out as described before with the following exceptions [21]. E2 samples for isothermal titration calorimetry (ITC) were subjected to an additional step of size-exclusion chromatography (SEC) in 1× ITC buffer (20 mM sodium phosphate, pH 8.0, and 150 mM NaCl) using a Superdex 75 pg 16/600 column (GE Healthcare Lifesciences, Sweden) soon after the ion-exchange chromatography. For the preparation of the Ube2N/Ube2V1 complex, individually purified protein samples after ion exchange were mixed in stoichiometric amounts and the complex was isolated using SEC in a Superdex 75 pg 16/600 column as described previously [22].

The first and the second steps employed for the purification of full-length ZNRF1 and its variants were identical with that of the E2s, except that all the buffers were supplemented with 20 µM ZnSO_4_. ZNRF1^CTD^ and ZNRF1^RING^ constructs were further purified using SEC in a Superdex 75 pg 16/600 column (GE Healthcare) after the second HisTrap column purification step. Full-length ZNRF1 was subjected to a third round of affinity chromatography using StrepTrap HP columns and eluted with 2 mM d-desthiobiotin in the equilibriation buffer. The protein was subsequently desalted in 1× ITC buffer (20 mM sodium phosphate, pH 8.0, and 150 mM NaCl) for isothermal titration calorimetry.

### Fluorescent labeling of Ub

Fluorescent-labeled Ub samples were prepared by covalently conjugating a mutant Ub construct (Ser20 → Cys) to thiol-reactive fluorescein 5′-maleimide. The Ub^S20C^ mutant, upon purification, was buffer exchanged into 20 mM sodium phosphate (pH 7.4) containing 0.1 mM of TCEP using a 5 ml desalting column (GE Healthcare Lifesciences, Sweden). Labeling was achieved by incubating the protein with a two-fold molar excess of freshly prepared fluorescein-5′-maleimide in DMSO (Thermo Pierce Scientific LLC, U.S.A.) at 25°C for 1 h in the dark. Unreacted dye molecules were quenched with the addition of 10 mM β-mercaptoethanol and removed from the protein using the 5 ml Hitrap desalting column equilibrated with 20 mM Tris–HCl (pH 8.0). The labeled protein was concentrated and stored at −80°C in small aliquots.

### Ubiquitination assays

All *in vitro* ubiquitination reactions were performed in an assay buffer containing 25 mM Tris–HCl (pH 7.6), 100 mM NaCl, 5 mM ATP, and 5 mM MgCl_2_ at 37°C unless stated otherwise. To monitor unanchored Ub chain synthesis by the Ube2N/Ube2V1 complex, 20 µl of reaction mixtures were assembled by adding 500 nM E2 complex with Ub^FL^ (20 µM) and ZNRF1^CTD^ or its mutants at indicated concentrations in the assay buffer. Reactions were initiated with the addition of 100 nM E1, incubated for the desired time intervals, and terminated with the addition of reducing gel loading dye (NuPAGE sample buffer containing 100 mM β-mercaptoethanol; Life Technologies, Inc., U.S.A.). Reaction mixtures were resolved by denaturing SDS–PAGE and subsequently imaged in a Typhoon Trio+ Imager (GE Healthcare Lifesciences, Sweden) using the 526SP emission filter. Quantitative estimations of ubiquitination were obtained by measuring the conjugated fluorescence as a fraction of the total using ImageJ.

### Size-exclusion chromatography

Analytical size-exclusion chromatographies to purify E3 : E2 complex or molecular mass estimations were carried out using a Superdex 75 10/300 GL column (GE Healthcare Lifesciences, Sweden) in an ÄKTA Avant 25 chromatography system (GE Healthcare Lifesciences, Sweden). The column was first calibrated with the gel filtration molecular mass marker kit (GE Healthcare Lifesciences, Sweden). Individual protein samples or mixtures were applied to the column pre-equilibrated with GF buffer [20 mM Na-HEPES (pH 7.7), 150 mM NaCl, and 0.5 mM DTT] and elution profiles were monitored by recording the absorbance at 280 nm.

### Isothermal titration calorimetry

Isothermal titration calorimetries (ITC) were performed on a VP-ITC microcalorimeter (MicroCal, Inc., Northampton, U.S.A.) or AffinityITC LV (TA Instruments, U.S.A.) at 25°C. All protein samples used in ITC experiments were subjected to a final purification step over a Superdex 75 pg 16/600 size-exclusion column equilibrated in 1× ITC buffer [20 mM sodium phosphate (pH 8.0) and 150 mM NaCl]. Typically, 10–30 µM of ZNRF1^CTD^ or its variants were loaded into the cell, and E2s were delivered by a series of 10–12 µl (VP-ITC) or 2 µl (AffinityITC LV) injections from the syringe with constant stirring at 310 or 75 rpm, respectively. Each of the injections was separated by 3-min intervals to allow the system to reach baseline. Raw data obtained after the titrations were processed in NanoAnalyze software from TA instruments using a single site-binding model consistent with the crystal structure.

### Crystallization and data collection

ZNRF1^CTD^ and Ube2N formed a stable 1 : 1 complex in SEC. Crystallization samples were therefore prepared by mixing the proteins at ∼1 : 1 ratio with slight access of ZNRF1^CTD^ followed by SEC using a Superdex 75 10/300GL column in GF buffer. ZNRF1^CTD ^: Ube2N co-crystals were grown at 20°C by hanging-drop vapor-diffusion using a 1 : 1 mixture of the protein complex (total concentration 8 mg/ml) and a crystallization cocktail containing 0.2 M magnesium formate dehydrate and 20% (w/v) PEG 3350. For data collection, all the crystals were flash-frozen in liquid nitrogen in the crystallization cocktail supplemented with 20% (v/v) glycerol. Diffraction data were collected at the PX beamline BL21 at the Indus Synchrotron (Indore, India).

**Table 1:** Crystallographic data collection and refinement

	ZNRF1^CTD^/Ube2N
Data collection	
Wavelength	0.979470 Å
Space group	*P*2_1_2_1_2_1_
Cell dimensions (in Å)	*a =* 47.43, *b =* 59.55, *c =* 94.79
Cell angles (in °)	*α = β *=* γ = *90
Resolution^1^ (in Å)	47.43–1.47 (1.50–1.47)
*R*_merge_^1^ (in %)	0.055 (0.872)
*R*_pim_^1^ (in %)	0.031 (0.587)
*I*/*σI*	20.6 (1.7)
Completeness^1^ (in %)	99.2 (89.9)
Anomalous completeness^1^ (in %)	98.3 (81.4)
Redundancy^1^	7.7 (5.3)
Structure refinement	
Resolution	47.43–1.47
No. of reflections	43 644
*R*_work_/*R*_free_	16.5/18.4
*B*-factor (average)	22.156 Å^2^
RMSD (bond length)	0.0056 Å
RMSD (bond angles)	1.4463°
Ramachandran details	240 (preferred), 5 (allowed)
Structure details	
Protein atoms	2071
Waters	269
Ions	3 (Zn^2+^)
Other hetero atoms	10 (polyethylene glycol)

1 Values in parentheses are for highest-resolution shell.

### Data processing and structure determination

Diffraction data were indexed and integrated using XDS followed by scaling and merging in AIMLESS/POINTLESS from the CCP4 software suite [23]. The anomalous signal of Zn^2+^ atoms was utilized for initial SAD phasing of the Ube2N : ZNRF1^CTD^ crystal using the SHELX suite [24] and the model was built using BUCCANEER [25]. Subsequent rounds of manual building and refinements were carried out using COOT [26], REFMAC5 [27], and Phenix.Refine [28].

## Results and discussion

### ZNRF1^CTD^ activates Ube2N/Ube2V1-mediated free chain synthesis and binds Ube2N with nanomolar affinity

**Figure 1.**
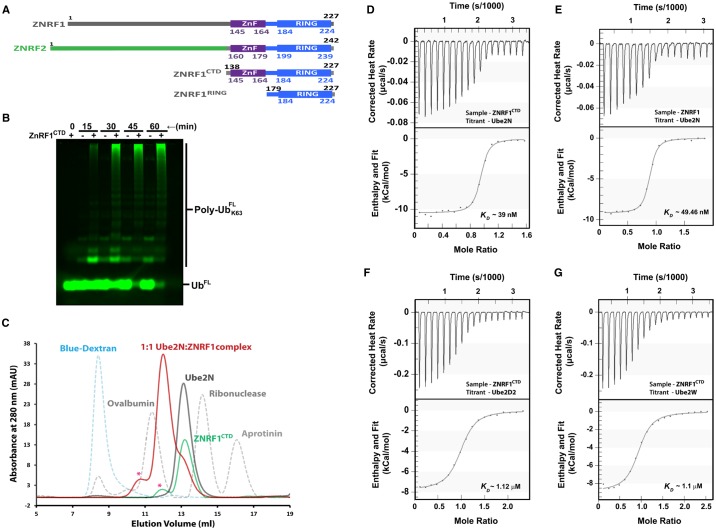
ZNRF1^CTD^ activates ubiquitination and uniquely binds Ube2N with nanomolar affinity. (**A**) Domain organization of ZNRF1, ZNRF2, and the truncated constructs designed for the present study. Color coding is used to indicate sequence similarity. (**B**) ZNRF1^CTD^ (500 nM) enhances Lys63-linked Ub chain synthesis by Ube2N/Ube2V1 (500 nM). Assay mixture also contains 100 nM Uba1, 25 µM Ub^FL^ in an assay buffer of pH 7.4 containing 5 mM ATP/Mg^2+^. Assays were carried out at 37°C for the indicated time intervals, stopped with SDS–PAGE sample buffer, and analyzed using SDS–PAGE followed by imaging in Typhoon Trio+. (**C**) Elution profiles of ZNRF1^CTD^, Ube2N, and their complex along with that of the standard molecular mass markers and blue dextran (indicated with colors) from a Superdex 75 10/300 GL column. Calculated molecular mass of the complex suggests ZNRF1^CTD^ to be monomer bound to a single Ube2N molecule. ‘*’ indicates a minor fraction of ZNRF1^CTD^ dimer present in the ZNRF1^CTD^ containing samples. (**D–G**) Binding isotherms for (**D**) ZNRF1^CTD ^: Ube2N, (**E**) ZNRF1 : Ube2N, (**F**) ZNRF1^CTD ^: Ube2D2, and (**G**) ZNRF1^CTD ^: Ube2W interactions obtained at 25°C using ITC. Titrations were carried out in an AffinityITC LV calorimeter in 20 mM Na-phosphate buffer (pH 8.0) containing 150 mM NaCl. Data were analyzed in NanoAnalyze software.

ZNRF1, along with its homolog ZNRF2, has been demonstrated to co-purify with Ube2N upon immunoprecipitation [14]. Such stable complex formations between E3s and E2s have often been observed when E3s contain additional E2-interacting domains, though no such Ube2N-interacting region has been yet been reported in any of its cognate E3s. To identify the presence of such a novel region in ZNRF1 and ZNRF2, if any, we compared their amino acid sequences. The comparison reveals that ZNRF1 and ZNRF2 share a highly conserved C-terminal region comprising the RING and the ZnF domains, while the N-terminal regions bear no significant resemblance (see Supplementary Figure S1). We, therefore, presumed that the C-terminal domains of these two E3s contain all the necessary elements for binding to Ube2N, while the diverse N-terminals only confer alternate substrate specificity. Accordingly, we prepared the C-terminal domain of ZNRF1, ZNRF1^CTD^, comprising residues 139–227 of the full-length protein (Figure 1A). The activity of this truncated construct was monitored by its ability to enhance Ube2N/Ube2V1-mediated Lys63-linked Ub chain synthesis in a multi-turnover assay (Figure 1B). Indeed, we find that ZNRF1^CTD^ can robustly enhance the Ube2N/Ube2V1-catalyzed unanchored chain synthesis similar to other Ube2N-interacting E3s, such as RNF8 and Rad5. We also find that ZNRF1^CTD^ could form a stable 1 : 1 complex with Ube2N to elute as a single species in SEC, suggesting high-affinity interaction between these two proteins (Figure 1C).

Next, we carried out isothermal titration calorimetry (ITC) experiments to quantitate the binding affinity between ZNRF1 and Ube2N. Experiments reveal that Ube2N binds ZNRF1^CTD^ with a dissociation constant (*K*_d_) of ∼39 nM at 25°C (Figure 1D). Interestingly, in contrast with other E3s, binding of ZNRF1 to Ube2N is driven by a favorable change in enthalpy (Δ*H*_binding_ ≈ −10.5 kcal/mol) with entropy (Δ*S*_binding_) played only a minor role (*T*Δ*S*_binding_ 0.4 kcal/mol at 25°C). Similar to Ube2N alone, calorimetric titrations of ZNRF1^CTD^ with the Ube2N/Ube2V1 complex yield a *K*_d_ of ∼39 nM at 25°C, suggesting against any role of Ube2V1 in E3 binding, as expected (Supplementary Figure S2A). We further tested the binding of Ube2N and full-length ZNRF1 to confirm against any role of the ZNRF1 N-terminal region in E2 binding. Indeed, under identical conditions, a ZNRF1 : Ube2N titration yields a similar *K*_d_ of ∼49 nM confirming our conjecture (Figure 1E).

We further extended our calorimetric investigations to measure the affinity of ZNRF1^CTD^ for Ube2D2 and Ube2W as ZNRF1/2 eluted with minor amounts of Ube2Ds, Ube2Es, and Ube2W in the co-immunoprecipitations besides Ube2N. Calorimetric studies yield *K*_d_s of ∼1.1 µM for both ZNRF1^CTD ^: Ube2D2 and ZNRF1^CTD ^: Ube2W complexes (Figure 1F,G). Such affinities between E3s and E2s have, however, been reported recently [6], confirming that the nanomolar affinity is unique for the ZNRF1 : Ube2N pair. Moreover, similar Ube2N affinity observed for the full-length ZNRF1 and ZNRF1^CTD^ allowed us to conclude against the presence of any additional E2-binding region in the full-length protein. We, therefore, restricted ourselves to ZNRF1^CTD^ for further analysis.

### ZNRF1^CTD^ binds Ube2N exclusively via its RING domain

**Figure 2.**
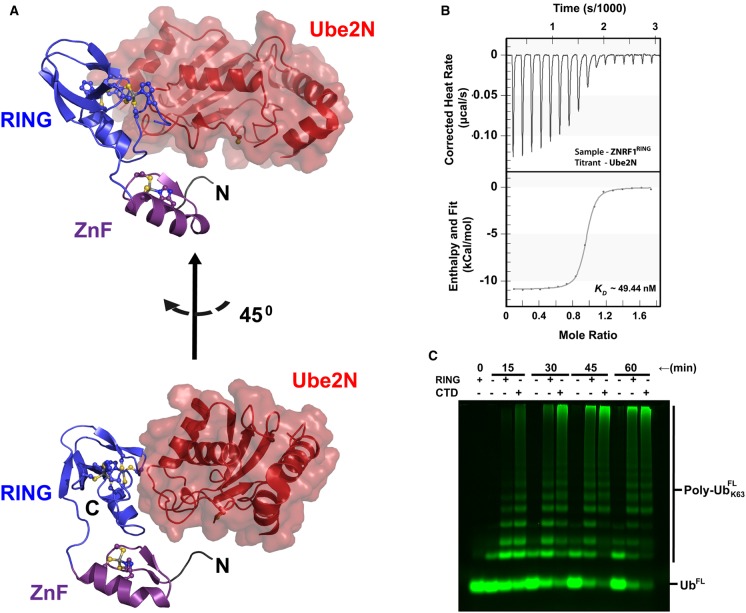
ZNRF1^CTD^ binds Ube2N exclusively via its RING domain. (**A**) Crystal structure of ZNRF1^CTD^ bound to Ube2N at 1.47 Å resolution. RING (blue) and ZnF (purple) domains are represented in cartoon, while Ube2N (red) is presented with semi-transparent surface and cartoon. Figure has been generated with the open source version of PYMOL. (**B**) Binding isotherm showing the titration of ZNRF1^RING^ with Ube2N. The titration and the data analysis are carried out identically as described in Figure 1. (**C**) A comparison of catalytic activities of ZNRF1^CTD^ and ZNRF1^RING^. Assays were done to monitor the Lys63-linked Ub-chain synthesis by Ube2N/Ube2V1 as in Figure 1B.

A comparison of the *K*_d_ for the ZNRF1 : Ube2N complex with that of all known E3–E2 complexes confirms its uniqueness (Supplementary Table S1). We set forth to determine the crystal structure of ZNRF1^CTD^ in complex with Ube2N to get the molecular details behind this high-affinity interaction between an E3 and its cognate E2. Crystallization trials were carried out with SEC-purified ZNRF1^CTD ^: Ube2N samples as it yielded homogenous preparations as opposed to mixing purified proteins in stoichiometric amounts. Crystals were successfully obtained in the *P*2_1_2_1_2_1_ space group and allowed us to collect a 1.47 Å dataset (Table 1). The structure solved using the anomalous signal from the Zn^2+^ atoms contains one molecule of ZNRF1^CTD^ in the asymmetric unit of the crystal together with one molecule of Ube2N bound to the RING domain in a canonical manner (Figure 2A). The ∼520 Å^2^ interface area of the ZNRF1 : E2 interface is also comparable to most RING E3 : E2 complexes solved till date (Supplementary Table S1). ZnF domain contributes minimally in engaging Ube2N in the canonical manner, but contacts a symmetry-related Ube2N molecule in the crystal facilitating crystal packing. This contact is not possible within the asymmetric unit protomers due to steric restrictions and does not also seem to be important for the affinity as ZNRF1^CTD^ in our ITC titrations never yielded any stoichiometry other than ∼1 : 1. Moreover, binding of ZnF to Ube2N as observed in our crystal occludes the activated Ub to adopt closed conformation required for the catalysis. To further confirm the dispensability of the ZnF domain in engaging the E2, we prepared the ZNRF1^RING^ construct encompassing residues 166–227 (Figure 1A) and subjected to ITC with Ube2N. As expected, ZNRF1^RING^ binds Ube2N with a *K*_d_ of ∼49 nM comparable to that of the Ube2N : ZNRF1^CTD^ and Ube2N : ZNRF1 complexes (Figure 2B). This truncated construct could also enhance free Ub chain synthesis by the Ube2N/Ube2V1 complex (Figure 2C). We, however, noted that ZNRF1^RING^ domain, unlike ZNRF1^CTD^, was quite unstable upon purification and often formed soluble aggregates incapable of binding to the E2 or enhancing Ub chain synthesis. Thus, it appears that the ZnF domain plays a role in the stability of ZNRF1. Interestingly, Traf6, another Ube2N-interacting E3, required the presence of one of its three ZnF domains in E2 binding [29]. Despite that, Traf6–Ube2N complex structure (PDB ID: 3HCT or 3HCU) failed to show any physical interaction between the E2 and ZnF domains. ZnF domain might also play some functional roles in the case of ZNRF1 as suggested for Traf6 [30]. In any case, both the crystal structure and ITC-binding experiments on ZNRF1 agree to confirm that this E3 binds Ube2N exclusively via its RING domain.

### Molecular basis of ZNRF1^CTD ^: Ube2N affinity

**Figure 3.**
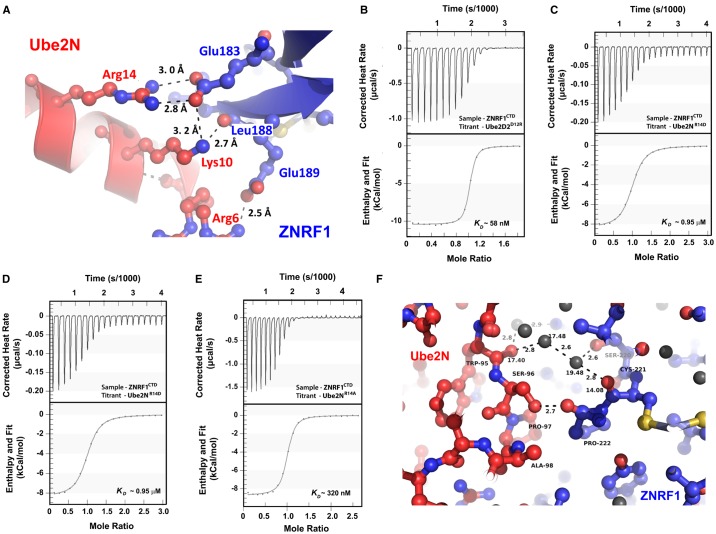
Contribution of Arg14^Ube2N^ : Glu183^ZNRF1^ H-bond/salt-bridge interaction in ZNRF1 : Ube2N affinity. (**A**) A close up of ZNRF1^CTD^ (in blue) : Ube2N (in red) interface depicting the H-bonding network involving Arg14, Lys10, and Arg6 of Ube2N with Glu183, Leu188, and Glu189 of ZNRF1. H-bonding distances are also indicated. (**B–E**) Binding isotherms of ZNRF1^CTD^ to Ube2D2^D12R^ (**B**), Ube2N^R14D^ (**C**), Ube2N^R14A^ (**D**), and that of ZNRF1^CTD^, E183A to wtUbe2N (**E**) at 25°C. Titrations and data analysis were carried out as in Figure 1. (**F**) ZNRF1^CTD^ and Ube2N also interact via water molecules forming an extensive network of H-bonds (shown with dotted lines). H-bonding distances are also mentioned. *B*-factors of the water molecules and that of the protein atoms are comparable illustrating their importance.

The absence of additional non-RING E2-interacting regions in ZNRF1 coupled with its high E2 affinity led to a detailed analysis of the ZNRF1^CTD ^: Ube2N interface. In fact, the best affinity between any RING E3 and its cognate E2 was reported for the highly specific FANCL : Ube2T complex (*K*_d_ ≈ 0.5 µM at 8°C), where the E3 and E2 buries a larger interface area of ∼700 Å^2^ unlike most E3 : E2 complexes including ZNRF1 : Ube2N [31,32]. We precisely looked for Ube2N-specific interactions by aligning Ube2D2 to our complex structure as the latter displayed ∼25-fold lower affinity. The analysis reveals that ZNRF1 : Ube2N interface contains three salt-bridges/H-bonds involving Glu183 of ZNRF1 and Arg14/Lys10 of Ube2N (Figure 3A). Ube2D2 cannot have two of these electrostatic interactions as it contains Asp (Asp12) in lieu of the Arg14 of Ube2N. We, therefore, swapped the Arg and Asp residues in both of the E2s to generate Ube2D2^D12R^ and Ube2N^R14D^ mutants and measured their affinities for ZNRF1^CTD^ using ITC. In perfect agreement with our structure, Ube2D2^D12R^ binds ZNRF1^CTD^ with an enhanced affinity (*K*_d_ ∼58 nM) similar to wtUbe2N, whereas Ube2N^R14D^ displays a *K*_d_ of ∼1 µM akin to wtUbe2D2 (Figure 3B,C).

As R14D mutation involved swapping of a charge, we tested the binding of ZNRF1^CTD^ to Ube2N^R14A^. In contrast with Ube2N^R14D^, Ube2N^R14A^ displays a nominal decrease in its ZNRF1^CTD^ affinity with a *K*_d_ of ∼320 nM at 25°C (Figure 3D). This change in the *K*_d_ upon replacing Arg with Ala corresponds to a loss of 1.2 kcal mol^−1^ in the free energy of complex formation (Δ*G*_binding_) and agrees with the expected value upon loss of two H-bonds. Similarly, mutating Glu183 in ZNRF1^CTD, E183A^ reduces its affinity for the wtUbe2N and yields a *K*_d_ of ∼0.5 µM that corresponds to an additional ∼0.4 kcal mol^−1^ in Δ*G*_binding_ compared with that of the wild-type E3 and Ube2N^R14A^ (Figure 3E). This greater change upon mutating the E3 is expected as it results in a loss of an additional H-bond mediated by Lys10 of Ube2N apart from the ones via Arg14. Nonetheless, based on our observations with Ala substitutions, we conjectured that the more pronounced effect of the Arg to Asp substitution in E2s occurred due to the introduction of electrostatic repulsion between the Asp of E2s with the Glu of ZNRF1. Thus, replacing the Arg with Glu instead of Asp in Ube2N is expected to cause a further increase in the repulsive forces presumably due to a decreased distance between the E2 side chain and Glu183 of the E3. Indeed, the Ube2N^R14E^ binds ZNRF1 with a *K*_d_ ∼1.1 µM supporting our hypothesis (Supplementary Figure S2B). This predominance of electrostatic interactions in ZNRF1 : Ube2N binding prompted us to carry out titrations at increasing salt concentrations of 300 and 450 mM (Supplementary Figure S2C,D). As expected, an increase in the ionic strength led to a concurrent decrease in *K*_d_ for ZNRF1/Ube2N binding by affecting the Δ*H*_binding_.

We further analyzed the role of two other Ube2N residues to delineate their role towards ZNRF1 affinity. These two residues, namely Ser96 and Ala98, are conserved across all ZNRF1-interacting E2s including Ube2D2. We chose Thr, a β-branched hydrophilic residue, to replace Ala98 to maximize the destabilizing effect as Ala98 binds to a hydrophobic pocket in ZNRF1. On the other hand, Ser96 was replaced with a long chain polar residue (Arg) to perturb the binding. Interestingly, both Ube2N^S96R^ and Ube2N^A98T^ could efficiently bind to ZNRF1^CTD^ albeit with lower affinity (*K*_d_ ≈ 203 and 408 nM for Ube2N^S96R^ and Ube2N^A98T^, respectively) (Supplementary Figure S2E,F). The A98T mutation destabilizes the E3 : E2 complex mostly via an unfavorable entropic change while the enthalpy contribution remains similar to that for the wild-type E2. S96R mutation, on the other hand, leads to a reduction in Δ*H*_binding_ along with a favorable entropic contribution (Δ*S*_binding_).

Besides these residues, our complex structure solved just shy of 1.5 Å, underscores the importance of several other H-bonding/salt-bridges between various side-chains and main-chain peptide moieties, water-mediated H-bonding interactions (Figure 3F), and hydrophobic residues such as Met64 (Supplementary Figure S3) in E3 : E2 interactions dictating the overall affinity. Hydrophobic interactions have been shown to play a major role in dictating the affinity between other E3 : E2 pairs. Mutating Phe63 in Ube2T, the residue that corresponds to Met64 in Ube2N, has been found to lead to an ∼10-fold reduction in FANCL binding [31], suggesting the importance of conserved hydrophobic interactions in dictating E2 : E3 affinity. In addition, the water-mediated H-bonding was particularly intriguing in our case as no such water-mediated bonds have been observed in any of the E3–E2 crystal structures. The crystallographic *B*-factor for the H-bonded water molecules at the interface is also comparable to that of the protein residues confirming their significant contribution in complex formation.

#### Implications of E2 affinities on the ubiquitination activity of ZNRF1

**Figure 4.**
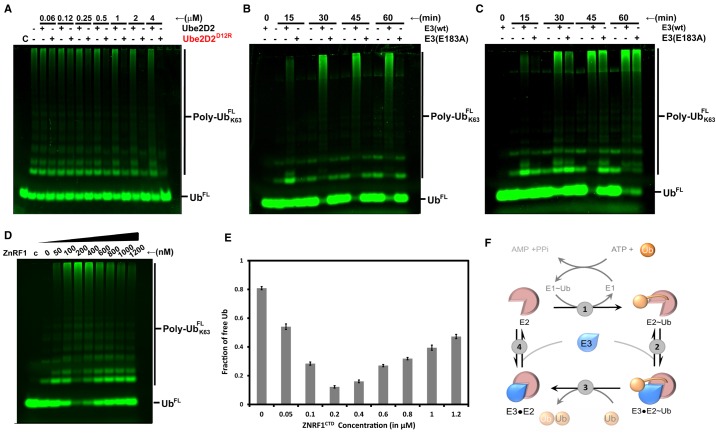
Functional implications of nanomolar affinity of ZNRF1 for Ube2N. (**A**) Ube2D2^D12R^, and not wtUbe2D2, can compete with Ube2N/Ube2V1 for ZNRF1^CTD^ under E3 limiting conditions. Assays were identical with those in Figure 1, except (i) E3 concentration was 50 nM, (ii) Ube2D2 or its mutant was added in increasing amounts as indicated in addition to 500 nM Ube2N/Ube2V1, and (iii) all incubations were of 15 min. Inhibition of Lys63-linked Ub chain synthesis upon the addition of Ube2D2^D12R^ is evident in the gel. (**B** and **C**) Synthesis of Lys63-linked Ub chains by Ube2N/Ube2V1 in the absence or in the presence of either 50 nM (**B**) or 500 nM (**C**) of ZNRF1^CTD^ or its E183A mutant as indicated. Assays were done as in Figure 1. Refer to the text for more details. (**D**) Inhibition of Ube2N/Ube2V1 activity with increasing concentrations of ZNRF1^CTD^. These fixed time point assays containing 500 nM of Ube2N/Ube2V1, 25 µM of Ub, and increasing concentrations of ZNRF1^CTD^ were incubated for 15 min at 37°C, stopped, and analyzed as in Figure 1. (**E**) Quantitative estimation of the free Ub remaining in the assay lanes of (**D**) as a fraction of the total. Densitometry has been carried out with ImageJ. Error estimates were obtained from experiments done in triplicates. (**F**) A schematic representation of the Ub cycle. At the steady state of Ub turn-over, the reaction rate is expected to be determined by the slowest step in the cycle. For E3s like ZNRF1 having high E2 affinity, dissociation in step (4) will act as the rate-determining step, particularly at comparable E3 : E2 concentrations. On the other hand, low E2 affinity of most E3s would allow them to follow the traditional enzyme kinetics.

Our binding experiments demonstrated that ZNRF1 binds Ube2D2 with ∼25-fold less affinity compared to Ube2N (Figure 1D,F). This difference in affinities suggests that ZNRF1, particularly at low concentrations, would preferentially interact with Ube2N over Ube2D2. In other words, under physiological condition, when both the E2s are present simultaneously, ZNRF1 is expected to modify its substrates by Ube2N/Ube2V1-mediated Lys63-linked chains as opposed to the non-specific chains built by Ube2D2. To validate this proposition *in vitro*, we deviced a competition assay where increasing concentrations of Ube2D2 was added in addition to 500 nM of the Ube2N/Ube2V1 complex to compete for 50 nM ZNRF1^CTD^ present in the reaction mixture. We could do this assay with active versions of both the E2s as Ube2D2 does not synthesize unanchored Ub chains. Moreover, Ube2D2 cannot ubiquitinate ZNRF1^CTD^ unlike the full-length protein due to the absence of a suitable acceptor lysine (data not included). Thus, in these modified assays, Ube2D2 and its Ub-thioester can potentially compete with Ube2N∼Ub/Ube2V1 for the E3 resulting in a slowdown of the Lys63-linked free-chain synthesis. The result shows no visible reduction in Ube2N/Ube2V1 activity at all wild-type Ube2D2 concentrations all the way up to 4 µM (Figure 4A). In contrast, increasing concentrations of Ube2D2^D12R^ progressively inhibits Ub chain synthesis by Ube2N/Ube2V1 leading to a complete inhibition at concentrations ≥1 µM (Figure 4A). The Ube2D2^D12R^ mutant can effectively compete out Ube2N, unlike the wild-type Ube2D2, due to its comparable ZNRF1 affinity for the latter (compare Figures 1D and 3B). Taken together, these data confirm our proposition that ZNRF1, at least under limiting concentrations, will preferably interact and activate its high-affinity partner, Ube2N/Ube2V1. However, an increase in the concentration of ZNRF1 can be expected to engage Ube2D2 leading to a change in the Ub-chain type. Furthermore, as Ube2D2-mediated ubiquitination leads to proteasomal degradation unlike Lys63-lined chains, this ‘E2 switching’ can potentially act as a proteostatic mechanism to regulate the *in vivo* concentration of ZNRF1. Interestingly, qualitative experiments have demonstrated similar promiscuous E2 interaction for quite a few E3 ligases [21,33], though the *in vivo* relevance of most of these interactions is yet to be worked out. Quantitative estimates of the E2 affinities for any of those promiscuous E3s are also not available. However, based on our ZNRF1 data, it appears that such concentration-based E2 switching mechanisms might also exist for other E3s and can act as a potential regulatory mechanism of E3 activity.

To probe the correlation between E3 : E2 binding affinity and activity further, we compared the ligase activities of ZRNF1^CTD^ and ZRNF1^CTD,E183A^ having ∼10-fold difference in their dissociation constants toward Ube2N. These assays were carried out at two different E3 concentrations of 50 nM (Figure 4B) and 500 nM (Figure 4C). Indeed, we observe that at 50 nM, these two E3s show drastic difference with the mutant being completely unable to enhance Lys63-linked chain synthesis (Figure 4B). On the other hand, at 500 nM, ZNRF1^CTD,E183A^ could efficiently activate Ub-chain synthesis reducing the difference between the wild type and mutant to a negligible level (Figure 4C).

The dependence of ubiquitination activity on the concentration of ZNRF1^CTD^ prompted us to probe into the effect of such changes in a systematic manner. We carried out a set of ubiquitination reactions for a fixed time interval of 15 min where the E1, Ub, and the E2 concentrations were kept identical while the concentration of ZNRF1^CTD^ was varied between 50 and 1200 nM (Figure 4D,E). As anticipated, increasing the ZNRF1^CTD^ concentration from 50 to 200 nM leads to a gradual increase in the Ub-chain synthesis compared with the no-E3 samples (compare lane 2 with lanes 3–5). Intriguingly, a further increase in the concentration of ZNRF1^CTD^ results in an inhibition of Ub-chain synthesis (lanes 7–10 in Figure 4D). Such an unusual observation, where an increase in the enzyme concentration leads to an inhibition of product formation occurs due to the high affinity of ZNRF1 for Ube2N and the multistep mechanism that underlies all ubiquitination reactions (Figure 4F). Ubiquitination reactions can be represented by a cycle, which is continuously fed with activated Ub by the E1 in step (1) that gets conjugated in step (3), upon which E2s and E3s dissociate to prepare for a fresh round of transfer. A high-affinity interaction between the E3 and unconjugated E2 (represented by step 4), particularly at comparable concentrations of the E3 and the E2, will result in the accumulation of substantial amount of unproductive E2 : E3 complex in the steady state leading to a concomitant decrease in the free E2 concentration. This sequestering of free E2 will slow down the formation of E2–Ub intermediates reducing the overall conjugation activity. It can be expected that similar E2 sequestration upon increasing E3 concentration can potentially be observed for all the E3 ligases. However, E3 ligases with a weak affinity toward uncharged E2s shall readily dissociate in step (4) or require biologically irrelevant concentrations to achieve similar levels of inhibition as the ZNRF1. Our data further indicate that an increase in the concentration of ZNRF1 can not only inhibit itself, but can potentially inhibit other Ube2N-dependent E3 ligases via E2 sequestration not only *in vitro* but also *in vivo*. It would, however, be unlikely under normal physiological conditions where E2 concentrations probably exceed that of the individual E3s. Taken together, it appears that a balance of E3 and the E2 enzymes are necessary for maintaining the desired functionality of the ubiquitination machinery and even a change in the concentration or affinity due to undesirable mutation of any of the components can perturb the balance leading to drastically altered consequences in cells.

## Conclusion

RING domain E3s most often interact with their E2 partners displaying a wide range of affinities in the micromolar to sub-millimolar range, unless assisted by additional E2-binding regions. Such low-affinity interactions between E3s and E2s seem to allow rapid recycling of E2s between E1 and the E3 during ubiquitination. ZNRF1 extends this range further to show that it is possible for RING domains to interact with their cognate E2s with affinities greater than 100 nM yet display robust ubiquitination activity. Given the fact that a large number of RING E3s are coded by eukaryotic genomes (≥600 in humans) with most being uncharacterized, ZNRF1/ZNRF2 may not be sole examples of such high-affinity E3 : E2 interaction.

Mutational analyses helped us to dissect the role of various Ube2N and ZNRF1 residues to show that additional electrostatic interactions apart from the conserved hydrophobic ones as in Ube2D2 are responsible for imparting unusually high affinity for this E3–E2 pair. A comparison of all the binding parameters (Table 2) shows that even the absence of ionic interactions mediated by Arg14 of Ube2N and Glu183A of ZNRF1 do not abrogate binding. Even increasing the ionic strength of our buffer conditions shows similar reductions in the dissociation constant, but fails to abolish the interaction. Thermodynamic parameters observed for the mutant proteins also corroborate with the importance of both electrostatic and hydrophobic effects in mediating ZNRF1 : Ube2N binding. Thus, mutating Arg14 in Ube2N or Glu183 in ZNRF1 altered the enthalpic contribution in binding (Δ*H*_binding_) while Δ*S*_binding_ remained relatively unaffected. On the other hand, mutating Ala98 in the hydrophobic core led to a more pronounced change in the Δ*S*_binding_ rather than a change in the enthalpy. Remarkably, the S96R mutant led to a drastic reduction in Δ*H*_binding_ which was compensated by a favorable Δ*S*_binding_. It is likely that the substitution of Ser with a longer side chain residue like Arg resulted in an enhanced solvent structuring in the unbound state of the E2. However, upon binding of E3, burial of the Arg releases these structured solvent molecules leading to a favorable change in the entropy. On the other hand, the increased bulkiness of Arg systematically perturbs close associations between other residues including polar groups leading to a decrease in Δ*H*_binding_. However, more experiments regarding the effect of this mutation will be required in the future to establish this conclusion.

**Table 2:** Thermodynamic parameters for the interaction of ZNRF1^CTD^ or its mutants with E2s and their mutants All the titrations were carried out at 25°C in 25 mM Na-phosphate (pH 8.0), 150 mM NaCl except #2 & #3, which were carried out at 300 and 450 mM NaCl concentrations. All titration data were fit to single site model using NANOANALYZE (TA Instruments, U.S.A.).

No.	Cell	Syringe	*K*_d_^1^ (M)	$\Delta G_{\mathrm{binding}}$ (kcal mol^−1^)	$\Delta H_{\mathrm{binding}}$ (kcal mol^−1^)	$\Delta S_{\mathrm{binding}}$ (kcal mol^−1^ K^−1^)	*n*
1	ZNRF1^CTD^	Ube2N	3.881 × 10^−8^	−10.12	−10.52	−1.384	0.915
2	-do-(300 mM)	-do-	1.712 × 10^−7^	−9.231	−9.630	−1.336	0.984
3	-do- (450 mM)	-do-	2.249 × 10^−7^	−9.070	−9.263	−0.649	0.960
4	-do-	Ube2D2	1.130 × 10^−6^	−8.113	−7.789	1.087	0.963
5	-do-	Ube2W	1.090 × 10^−6^	−8.158	−8.852	−2.406	0.906
6	ZNRF1^FL^	Ube2N	4.946 × 10^−8^	−9.967	−9.173	2.662	0.842
7	ZNRF1^RING^	Ube2N	4.944 × 10^−8^	−9.967	−10.92	−3.199	0.913
8	ZNRF1^CTD^	Ube2N/V1^2^	3.921 × 10^−8^	−10.10	−10.21	−0.338	1.044
9	-do-	Ube2N^R14D^	9.480 × 10^−7^	−8.217	−8.383	−0.555	0.966
10	-do-	Ube2D2^D12R^	5.798 × 10^−8^	−9.873	−10.45	−1.953	0.976
11	-do-	Ube2N^R14A^	3.199 × 10^−7^	−8.861	−8.694	0.559	0.958
12	-do-	Ube2N^R14E^	1.078 × 10^−6^	−8.141	−8.542	−1.344	0.854
13	-do-	Ube2N^S96R^	2.030 × 10^−7^	−9.130	−5.644	11.69	0.828
14	-do-	Ube2N^A98T^	4.081 × 10^−7^	−8.716	−9.615	−3.013	1.048
15	ZNRF1^CTD, E183A^	Ube2N	4.594 × 10^−7^	−8.646	−9.416	−2.582	0.848

1 Dissociation constant.

2 Ube2N/Ube2V1 complex.

Our biochemical data underscore the importance of carrying out ubiquitination assays at different concentrations of the components to get a clearer understanding of activity and effect of mutations on E3 or E2s or both. This is illustrated by our studies on ZNRF1^CTD^ and ZNRF1^CTD,E183A^, where a comparison carried out at either 50 or 500 nM E3 would have led to quite different interpretations while correct interpretation required experiments at both the concentrations. The most important consequence of the unusual E2 affinity of ZNRF1 lies in the fact that an increase in its concentration leads to the sequestration of its preferred E2 partner slowing down the most relevant ubiquitination pathway instead of augmenting it. Such an aberrant increase in the ZNRF1 concentration may additionally promote alternate modification topologies mediated by other ‘less preferred E2s’ such as Ube2D2. Furthermore, being part of a network of interconnected pathways, sequestration of a given E2 *in vivo* may lead to abnormal changes in the cellular physiology by affecting other pathways dependent on it. Taken together, it seems likely that the overexpression of ZNRF1 or E3s with similarly high E2 affinity commonly done for functional studies might lead to the discovery of a less relevant biological phenomenon than originally intended and therefore must be approached with caution. For E3s with low E2 affinity, we do not expect such anomalies. Nonetheless, our findings reported in the present study apart from providing the detailed study on an E3 : E2 pair underscores the importance of carrying out detailed *in vitro* studies to design and execute better experiments to decipher the biology.

## Abbreviations

CTD: C-terminal domain
HECT: homologous to E6-AP C-terminus
IPTG: isopropyl-1-thio-β-d-galactopyranoside
ITC: isothermal titration calorimetry
RBR: RING between RINGs
RING: Really Interesting New gene
SEC: size-exclusion chromatography
Ub: ubiquitin
ZnF: zinc finger domain
