# Supplementary material for: Structural insights into the nanomolar affinity of RING E3 ligase ZNRF1 for Ube2N and its functional implications
Source: Biochem J. 2018 May 9;475(9):1569–82. doi: 10.1042/BCJ20170909 (PMC5941314; doi:10.1042/BCJ20170909)
Supplement: Supplementary Figures and Table [file BCJ-475-1569-s1.pdf]

Supplementary Figure S1.

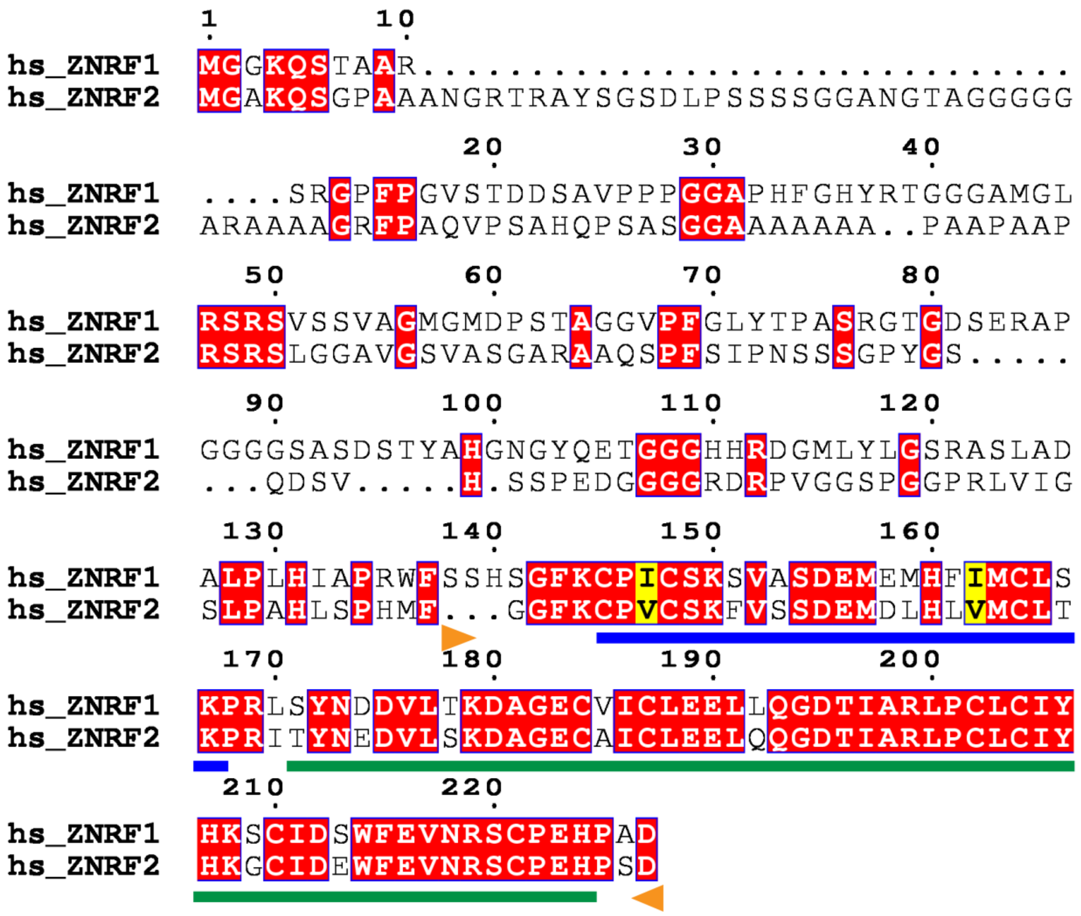

Figure S1. Clustal Omega alignment of ZNRF1 and ZNRF2 sequences.

Sequences of human ZNRF1 (Uniprot ID: Q8ND25) and ZNRF2 (Uniprot ID: Q8NHG8) were aligned using Clustal Omega and colored using ESPRIPT. Conservation of the C-terminal domain containing the ZnF (underlined in blue) and the RING (underlined in green) domains between these two E3s can be seen. Orange arrows denote the positions corresponding to the N-terminal and C-terminal ends of the ZNRF1^{CTD} construct.

Supplementary Figure S2.

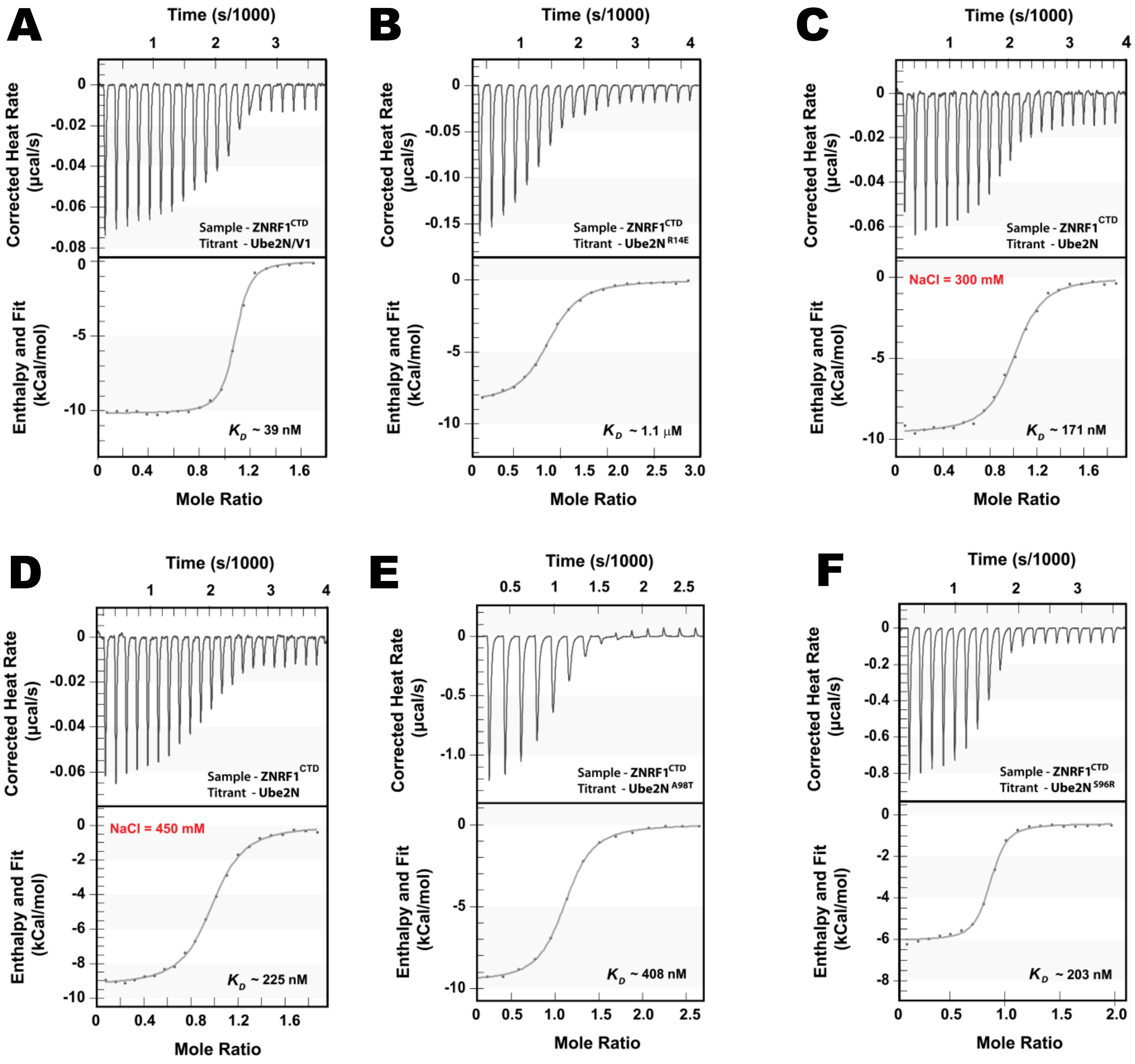

Figure S2. ITC binding isotherms.

ITC binding data for various E2 mutants and at increased salt concentrations as indicated. All the titrations were performed in 20 mM Na-phosphate buffer (pH 8.0) containing 150 mM NaCl except panels (C) and (D), where NaCl concentrations were raised to 300 mM and 450 mM respectively. Titrations were carried out either in an AffinityITC LV (TA instruments, USA) or VP-ITC calorimeter (Microcal Inc., USA) at 25°C (see methods for details). Dissociation constants were calculated by fitting the binding isotherms with NanoAnalyze software (TA instruments, USA). Please refer to Table 2 in the main text for binding parameters.

Supplementary Figure S3.

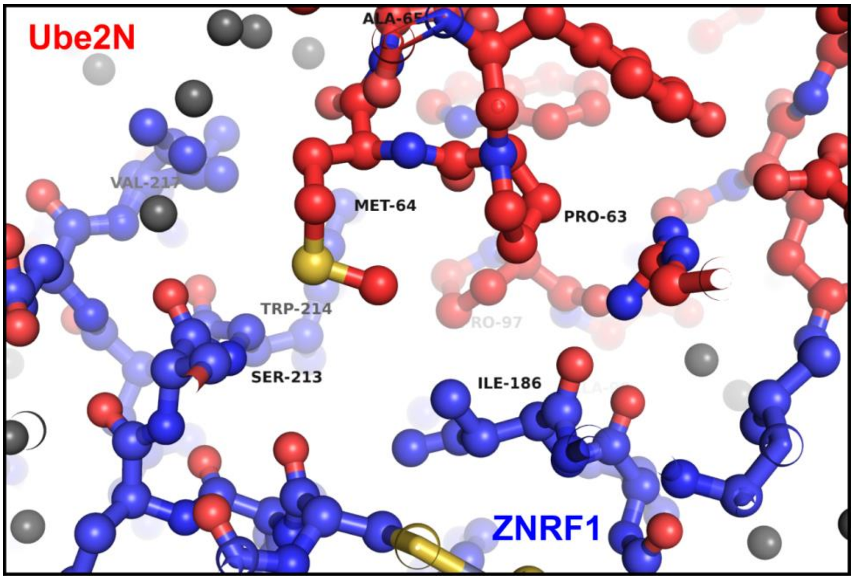

Figure S3. A close-up view of the ZNRF1:Ube2N Interface

Ball-and-stick representations of the ZNRF1^{CTD} (Blue) and Ube2N (red) interface. Water molecules are depicted as grey spheres. Hydrophobic residues pack intimately against each other in the ZNRF1^{CTD}:Ube2N interface and occludes any bound water molecules in the core.

Table S1: A comprehensive list of various E3:E2 pairs and their binding affinities

Pdb ID	E3	E2	K_d	Interface area (Å^2)*	Temp (°C)	Technique	NR†	PMID └
4AP4	RNF4	Ube2D1	15 µM[1]	520	10	AUC		27678051
4QPL	RNF146	„	----	680				25327252
5FER	TRIM25	Ube2D2	----	527				27154206
4V3K, 4V3L	RNF38	„	89±1 µM	525	25	SPR		25801170
4AUQ	BIRC7	„	NM [2]	445	25	SPR		22902369
3EB6	cIAP2	„	19-43 µM	591	25	ITC		18784070
4A49	cbl	„	42±2 µM	680	25	SPR		18996392
5MNJ	MDM2	„	----	485				28553961
5D0M	RNF165	„	~1 µM	525	25	ITC		26656854
5VZW	TRIM23	„	----	480				28681414
5D1K	RNF25^{RING}	„	14±1 µM	610	25	MT		26475854
5D1K	RNF25	„	180±32 nM	1545			YES	26475854
4S3O	RING1b	Ube2D3	6.1±0.4 µM	540		BIL		26151332
3RPG	BMI1	„	5±1 µM	610	30	BIL		21772249
4LAD	Gp78C	Ube2G2	3 nM	1165			YES	23942235
4CCG	FANCL^{RING}	Ube2T	454±128 nM	732	8	ITC		24389026
5EYA	TRIM25	Ube2N	----	545				27425606
3HCT, 3HCU	TRAF6	„	1.7±0.04 µM	555	25	SPR		19465916
4ORH	RNF8	„	1.6 µM	749		SPR		22589545
	ZNRF1	„	40 nM	520	25	ITC		This study

*** = Average interface area in Å^2(approximated to the next number); [1] K_d is determined for E2D2,**
[2] NM = not measurable, "----" indicates no K_d is reported
† NR= non-RING Elements; PMID └ = Hyperlinked pubmed entry for the article containing the data.
All outliers in terms of K_d and experimental temperature are highlighted in red.

MT = Microscale thermophoresis, SPR = Surface Plasmon Resonance, ITC= Isothermal Titration Calorimetry, BIL = Bilayer Interferometry
